# Impact of Preexisting Interstitial Lung Disease on Acute, Extensive Radiation Pneumonitis: Retrospective Analysis of Patients with Lung Cancer

**DOI:** 10.1371/journal.pone.0140437

**Published:** 2015-10-13

**Authors:** Yuichi Ozawa, Takefumi Abe, Minako Omae, Takashi Matsui, Masato Kato, Hirotsugu Hasegawa, Yasunori Enomoto, Takeaki Ishihara, Naoki Inui, Kazunari Yamada, Koshi Yokomura, Takafumi Suda

**Affiliations:** 1 Department of Respiratory Medicine, Respiratory Disease Center, Seirei Mikatahara General Hospital, Hamamatsu, Japan; 2 Department of Radiation Oncology, Seirei Mikatahara General Hospital, Hamamatsu, Japan; 3 Department of Clinical Pharmacology and Therapeutics, Hamamatsu University School of Medicine, Hamamatsu, Japan; 4 Second Division, Department of Internal Medicine, Hamamatsu University School of Medicine, Hamamatsu, Japan; University of Maastricht (UM), NETHERLANDS

## Abstract

**Introduction:**

This study investigated the clinical characteristics and predictive factors for developing acute extended radiation pneumonitis with a focus on the presence and radiological characteristics of preexisting interstitial lung disease.

**Methods:**

Of 1429 irradiations for lung cancer from May 2006 to August 2013, we reviewed 651 irradiations involving the lung field. The presence, compatibility with usual interstitial pneumonia, and occupying area of preexisting interstitial lung disease were retrospectively evaluated by pretreatment computed tomography. Cases of non-infectious, non-cardiogenic, acute respiratory failure with an extended bilateral shadow developing within 30 days after the last irradiation were defined as acute extended radiation pneumonitis.

**Results:**

Nine (1.4%) patients developed acute extended radiation pneumonitis a mean of 6.7 days after the last irradiation. Although preexisting interstitial lung disease was found in 13% of patients (84 patients), 78% of patients (7 patients) with acute extended radiation pneumonitis cases had preexisting interstitial lung disease, which resulted in incidences of acute extended radiation pneumonitis of 0.35 and 8.3% in patients without and with preexisting interstitial lung disease, respectively. Multivariate logistic analysis indicated that the presence of preexisting interstitial lung disease (odds ratio = 22.6; 95% confidence interval = 5.29–155; *p* < 0.001) and performance status (≥2; odds ratio = 4.22; 95% confidence interval = 1.06–20.8; *p* = 0.049) were significant predictive factors. Further analysis of the 84 patients with preexisting interstitial lung disease revealed that involvement of more than 10% of the lung field was the only independent predictive factor associated with the risk of acute extended radiation pneumonitis (odds ratio = 6.14; 95% confidence interval = 1.0–37.4); *p* = 0.038).

**Conclusions:**

Pretreatment computed tomography evaluations of the presence of and area size occupied by preexisting interstitial lung disease should be assessed for safer irradiation of areas involving the lung field.

## Introduction

Classic radiation pneumonitis (cRP) clinically emerges 3–4 months after radiotherapy (RT), and it is restricted to the irradiated area. The dose and area of irradiation have been demonstrated to be related to the severity of cRP, and the proportion of the total lung volume irradiated with >20 Gy (V20) or >30 Gy (V30) and the mean lung dose (MLD) are widely used as predictive markers of symptomatic cRP [1,2]. Being different from cRP, cases of acute radiation pneumonitis, which develop within a few days or weeks after chest irradiation with new-onset bilateral extensive ground glass opacity or infiltration, have been reported [3,4,5,6,7,8,9,10,11]. Such cases of acute extended radiation pneumonitis (AERP) have been reported using non-standardized definitions and names such as extensive acute lung injury, severe radiation pneumonitis, acute respiratory distress syndrome, or acute exacerbation of interstitial lung disease (ILD), and this condition has remained unexplored collectively.

ILD, including pulmonary fibrosis, has been repeatedly reported to be associated with the risk of lung cancer [12,13,14]. Based on our previous study, the cumulative incidence of lung cancer in patients with idiopathic pulmonary fibrosis is 3.3% after 1 year and 15.4% after 5 years [15], and it is not rare to find preexisting ILD (pre-ILD) in patients with lung cancer. Several studies previously revealed that the presence of pre-ILD is a significant risk factor for severe radiation pneumonitis [4,11,16,17,18,19,20,21,22]. However, most of these studies defined radiation pneumonitis as a shadow restricted to the irradiated area or did not refer to the extent of radiological findings. To our knowledge, only Makimoto et al. defined “severe radiation pneumonitis” as a shadow expanded out of the irradiated area and explored risk factors, identifying the presence of pre-ILD as a significant risk factor for “severe radiation pneumonitis.” However, this study included only 111 patients, and it did not evaluate the radiological features of pre-ILD [4].

Of numerous types of ILD, usual interstitial pneumonia (UIP) patterns on chest computed tomography (CT) are reported to be associated with the risk for acute exacerbation of ILD in several conditions. According to Kenmotsu et al., patients with UIP-pattern ILD on chest CT had a higher frequency of acute exacerbation of ILD than those with non-UIP-pattern ILD (30% *vs*. 8%, *p* = 0.005) [23]. Regarding pulmonary resection, Sugiura et al. reported that 6/49 (13.6%) patients with typical honeycombing, which is reminiscent of the UIP pattern [24], as detected by chest CT, experienced acute exacerbation, in contrast to 0/83 patients (0%) without honeycombing [25]. Although these findings indicated the importance of the pretreatment evaluation of pre-ILD by chest CT, there is little information regarding the association between CT findings of pre-ILD and radiation-associated lung injury. Therefore, in this study, we investigated the clinical characteristics and predictive factors of AERP with a focus on the presence and pretreatment chest CT findings of pre-ILD.

## Patients and Methods

### Patient population

From May 2006 to August 2013, we retrospectively reviewed the clinical records of patients with lung cancer who received irradiation at our facility with a curative or palliative intent. Of 1429 irradiations occurring from May 2006 to August 2013, 651 involved irradiation of areas including the lung field with chest CT images taken within 6 months prior to irradiation that were available for evaluation, and thus, patients involved in these irradiations were eligible for inclusion in the current study. For the determination of irradiation of areas including the lung field, we first selected cases involving irradiation of the lungs, mediastinum, thoracic spine, costal bone, chest wall, pleura, breast bone, and scapula and subsequently reviewed the 3-dimensional treatment plans. Medical records were reviewed, and clinical, laboratory, and radiological findings before and after irradiation were collected. The current study was approved by the ethics committee of Seirei Mikatahara General Hospital (#14–3). All clinical investigations were performed according to the principles expressed in the Declaration of Helsinki. The data were collected and analyzed anonymously prior to reporting.

### RT and dosimetric parameters

From 2006 to 2010, an integrated RT system, including a 3-dimensional RT treatment planning machine (ECLIPSE Ver. 7.3, Varian Co, CA, USA) and linear accelerator (CLINAC 21EX, Varian Co.), were used for RT. The beam energy was 4 or 10 MV, and RT was prescribed at the isocenter using the Batho Power Law as the calculation algorithm. The treatment planning was based on 5-mm-thick and 5-mm interval CT scans obtained in the treatment position. After 2010, Novalis-Tx (Brain LAB AG, Feldkirchen, Germany) and ECLIPSE Ver. 8.9 were installed and employed, and they used a beam energy of 6 MV. Tissue heterogeneity correction using the analytical anisotropic algorithm was applied. The treatment planning was based on 2.5-mm-thicks and 2.5-mm interval CT scans obtained in the treatment position.

To investigate V20, V30, the lung volume spared from receiving a dose greater than 5 Gy (VS5), and MLD, a dose-volume histogram was calculated directly from the physical dose distribution with preserved data. The total lung volume was defined as the volume of both lungs minus the gross tumor volume. No adjustment for fraction size was performed. Dosimetric parameters are summarized in Table 1.

**Table 1:** Clinical Background of All Evaluated Patients.

	All (n = 651)	ILD(+) (n = 84)	ILD(−) (n = 567)	*p*-value
	Median (range)	Median (range)	Median (range)	
Age, years	71 (27, 93)	76 (57, 88)	70 (27, 93)	0.002*
Sex, male [n (%)]	523 (80.3)	81 (96.4)	442 (78.0)	<0.001*
Pack-year smoking	41 (0, 240)	50 (0, 174)	40 (0, 240)	<0.001*
Smoking history, yes [n (%)]	531 (81.6)	77 (91.7)	454 (80.1)	0.006*
Concurrent ChT, yes [n (%)]	228 (35.0)	21 (25.0)	207 (36.5)	0.039*
PS, 0 or 1 [n (%)]	470 (72.2)	53 (63.1)	417 (73.5)	0.046*
**Types of cancer [n (%)]**				0.0028*
Squamous cell	175 (26.9)	36 (42.9)	139 (24.5)	
Adenocarcinoma	256 (39.3)	20 (23.8)	236 (41.6)	
Small cell	85 (13.1)	13 (15.5)	72 (12.7)	
Others	31 (4.8)	5 (6.0)	26 (4.6)	
Unknown	104 (16.0)	10 (7.1)	94 (16.6)	
**Pulmonary function**				
% FVC	88.3 (35.4, 143)	85.1 (35.4, 115)	89.5 (36.9, 143)	0.024*
% FEV1.0	80.1 (19.1, 141)	78.9 (33.3, 116)	80.3 (19.1, 141)	0.423
% DLCO	91.6 (29.2, 166.2) (n = 232)	79.2 (29.2, 128.0) (n = 30)	93.4 (43.9, 166.2) (n = 202)	0.0157*
**Laboratory findings**				
LDH, IU/l	208 (78, 5874)	225 (123, 5874)	204 (78, 2882)	0.0013*
CRP, mg/dl	0.50 (0, 30.6)	1.2 (0, 21.6)	0.5 (0, 30.6)	0.0031*
**Radiotherapy**				
Total dose, Gy	50.0 (3.0, 72)	50 (6.0, 72)	50 (3.0, 70)	0.908
Dose per fraction, Gy	3.0 (1.5, 10)	2.1 (1.5, 10)	3.0 (1.5, 10)	0.252
V20, %	8.1 (0.0, 36.3)	8.7 (0, 36.3)	8.0 (0, 34.4)	0.985
V30, %	4.8 (0.0, 30.2)	5.3 (0, 28.5)	4.8 (0. 30.2)	0.595
VS5, %	80.8 (45.8, 100)	80.4 (45.8, 100)	80.8 (48.1, 100)	0.812
MLD, Gy	5.0 (0.2, 21.6)	5.0 (0.2, 17.6)	5.0 (0.2, 21.6)	0.786
Curative [n (%)]	376 (57.8)	46 (54.8)	330 (58.2)	0.552
**Target** organ [n (%)]				0.271
Lung/mediastinum	478 (73.4)	68 (81.0)	410 (72.3)	
Thoracic spine	121 (18.6)	9 (10.7)	112 (19.8)	
Costal bone	47 (7.2)	4 (4.8)	43 (7.6)	
Chest wall/pleura	17 (2.6)	3 (3.6)	14 (2.4)	
Others	5 (0.8)	0	5 (0.9)	

*, *p* < 0.05.

ILD, interstitial lung disease; ChT, chemotherapy; PS, WHO performance status; FVC, forced vital capacity; FEV, forced expiratory volume; DLCO, diffuse lung capacity of carbon monoxide; LDH, lactate dehydrogenase; CRP, C-reactive protein; V20_,_ volume receiving >20 Gy; V30_,_ volume receiving >30 Gy; VS5_,_ volume spared from 5 Gy; MLD, mean lung dose

### Diagnosis and scoring of ILD by pretreatment CT images

In total, 2 radiologists and 3 physicians specializing in pulmonology independently evaluated CT scans obtained within 6 months prior to irradiation. The images had been acquired with an axial slice thickness of 3–5 mm. Images with 3 mm thickness were available for 612 cases (94%).

Bilateral independent ground-glass abnormalities, reticular abnormalities, traction bronchiectasis, non-emphysematous cysts, and honeycombing were defined as findings indicative of ILD [24,26]. All patients were classified as having definite or possible ILD or no suspicion of ILD according to the CT findings. Definite ILD was defined as having one or more definite ILD-indicative findings, whereas no suspicion of ILD was defined by the absence of any such findings. The term “possible ILD” was allowed when the judges were unable to establish clear distinctions. Three or more concordant classifications were accepted as final. When only 2 assessors reached an agreement, the more severe category was adopted as the final judgment. Patients judged as having definite or possible ILD were considered to have pre-ILD in subsequent analyses.

**Fig 1:**
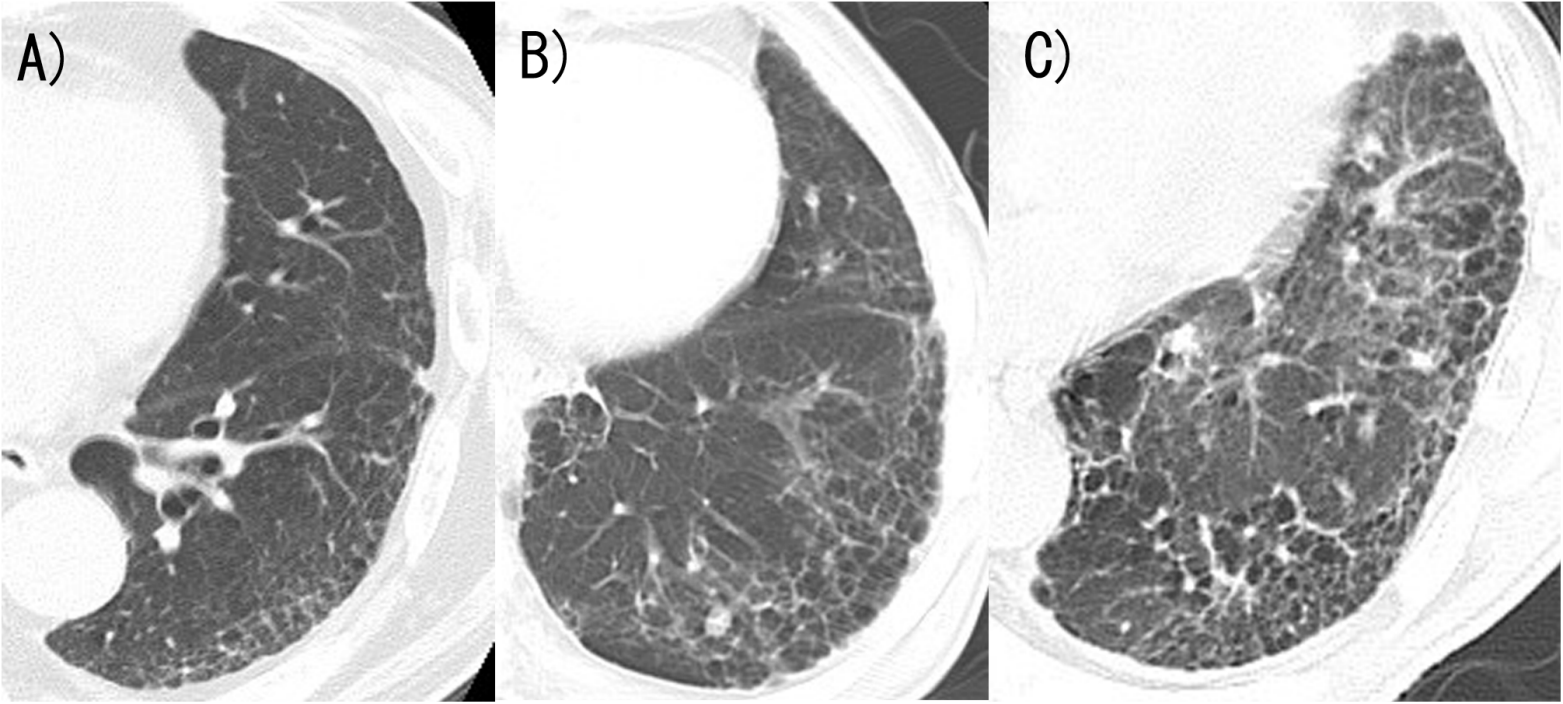
Representative chest computed tomography image of the area used for assessing the presence of preexisting interstitial lung disease. A), B), and C) were scored as 0–10, 10–25, and 25–40%, respectively.

In patients with pre-ILD, the radiological finding of ILD was measured by the same 5 specialists. Specifically, the lung area affected by pre-ILD was estimated and classified into 4 grades based on the CT findings as follows: 0–10%, 10–25%, 25–40%, and >40% (Fig 1). Furthermore, compatibility with the UIP pattern was evaluated according to the American Thoracic Society/European Respiratory Society consensus statement of 2011 [27]. According to the recommendation, we ranked all pre-ILDs into 1 of 3 grades: definite UIP, possible UIP, and inconsistent with UIP. All ratings were completed independently without any preliminary knowledge about the patients or other specialists’ decisions.

### Definition of AERP

We defined AERP according to the following features: (1) bilateral pulmonary ground-glass or infiltrative shadow that extended out of the irradiated area on both sides; (2) newly emerged during the course of or within 30 days after the completion of irradiation; and (3) absence of other explainable causes excluding irradiation, including pulmonary infection and congestive heart failure. Cases of pulmonary infection were excluded from the study based on the results of blood tests, sputum, and/or blood culture and the response to antibiotics.

### Statistical analysis

In patients with and without pre-ILD, clinical characteristics and treatment-related factors including age, sex, smoking status, concurrently administered chemotherapy, World Health Organization performance status (PS), baseline pulmonary function test (% vital capacity [VC]), forced expiratory volume in 1 s (FEV1.0), % diffuse lung capacity of carbon monoxide (% DLCO), and pretreatment serum lactate dehydrogenase (LDH) and serum C-reactive protein (CRP) levels were compared using the χ^2^ or Mann–Whitney *U* test. To investigate predictive factors, univariate and multivariate analyses were performed with logistic regression models using the following factors: age, sex, pack-year smoking, concurrent systemic chemotherapy, PS (0 or 1 *vs*. ≥2), irradiation dose per fraction, presence of pre-ILD, area occupied by pre-ILD (<10% *vs*. ≥10%), UIP compatibility of pre-ILD (definite or possible UIP *vs*. inconsistent with UIP), purpose of irradiation (radical *vs*. palliative), target organ (lung or mediastinum *vs*. others), pretreatment %FVC, pretreatment FEV1.0, and pretreatment serum LDH and CRP levels. The total irradiated dose, the mean lung dose, V20, V30, and VS5 were excluded from predictive factor analysis because irradiation was terminated in 5/9 patients with AERP because AERP developed in the middle of RT. The % DLCO was also excluded from the analysis because of the limited number of patients with available data (n = 232). Factors with a probability (*p*) value < 0.05 in univariate analysis were included in the multivariate analysis. For all analyses, *p* values were 2-sided, and *p* < 0.05 was considered statistically significant. All statistical analyses were performed using the PASW Statistics version 18.0 for Windows software (SPSS Inc., Chicago, IL, USA).

## Results

### Background of patients and pre-ILD

The clinical backgrounds of all analyzed patients are shown in Table 1. A total of 84 patients (13%) had pre-ILD. Patients with pre-ILD were significantly older (76 years *vs*. 70 years, *p* = 0.002), more commonly male (96% *vs*. 78%, *p* < 0.001), more commonly had a history of smoking (92% *vs*. 80%, *p* = 0.006), and less commonly had a PS of 0 or 1 (63% *vs*. 74%, *p* = 0.046). As predicted, %VC (85.1% *vs*. 89.5%, *p* = 0.024), % DLCO (n = 232) (79.2% vs. 93.4%, p = 0.0157) and LDH levels (225 *vs*. 204, *p* = 0.0013) were also significantly different compared with those in patients without pre-ILD; however, there was no statistical difference in irradiation including the total dose, target organs, or dosimetric parameters (V20, V30, VS5, and MLD). TheV20, V30, and MLD were comparatively low because of the high ratio of palliative RT. In total, 42% of patients (275 patients) received RT for the purpose of palliation. 24% of patients (155 patients) received stereotactic irradiation. Evaluation of pretreatment chest CT revealed that 81% of patients (68 patients) with pre-ILD had less than 10% lung involvement, and 14 (12 patients) and 5% of patients (4 patients) were estimated to have 10–25 and 25–40% involvement, respectively. No patients had pre-ILD involving more than 40% of the lungs. Regarding UIP compatibility, 8 (7 patients), 81 (68 patients), and 11% (9 patients) of the patients with pre-ILD were estimated to have a definite UIP pattern, a possible UIP pattern, and an inconsistent with UIP pattern, respectively.

### Clinical characteristics of AERP

**Fig 2:**
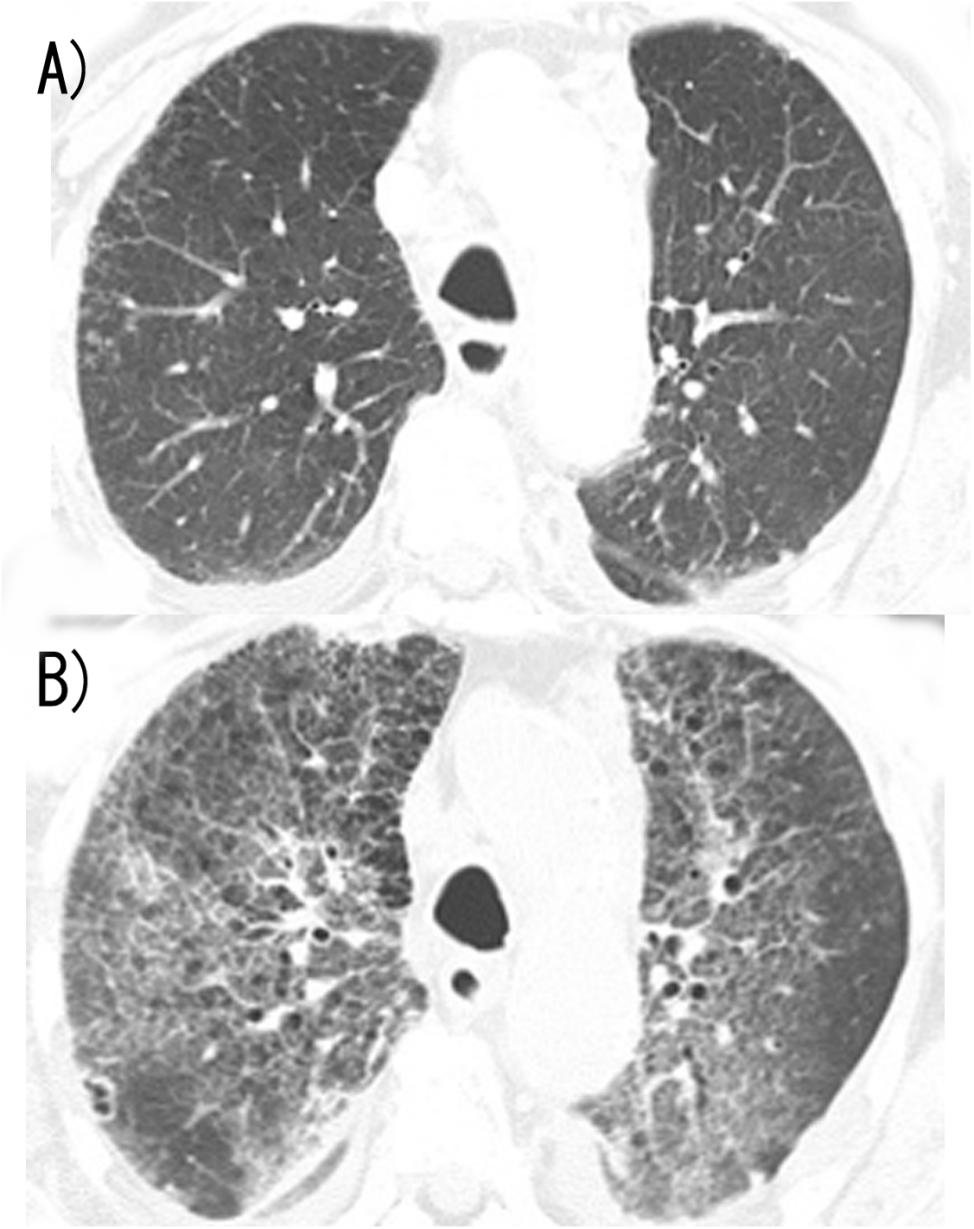
Chest computed tomography (CT) image of case no. 6. A) CT image obtained 7 days before irradiation to the thoracic spine showing a mild sub-pleural interstitial shadow and emphysema. B) CT image showing bilateral extended ground-glass abnormality superimposed on the pretreatment interstitial shadow.

In total, 9 of 651 patients developed AERP (1.4%). Representative CT images and detailed clinical data are shown in Fig 2 and Table 2. All patients with AERP were male, and the mean patient age was 71.6 years. Six patients developed AERP following palliative RT. No cases of AERP were caused by stereotactic irradiation Seven patients with AERP had pre-ILD; 2 and 5 of these patients were evaluated as having definite and possible UIP patterns, respectively. The percent area occupied by ILD was 0–10% in 2 patients, 10–25% in 4 patients, and 25–40% in 1 patient.

**Table 2:** Individual Data of Patients with AERP.

No.	Age (yr)	Sex	pre-ILD	Area of pre-ILD (%)	UIP compatibility of pre-ILD	Days to AERP	Concurrent chemotherapy	Outcome at 90 days after final RT
1	60	M	yes	25–40	definite	0*	no	dead
2	77	M	yes	10–25	definite	10	no	dead
3	72	M	yes	10–25	possible	0*	no	dead
4	58	M	yes	10–25	possible	21	cisplatin/vinorelbine	alive
5	80	M	yes	10–25	possible	0*	no	alive
6	85	M	yes	<10	possible	16	no	dead
7	69	M	yes	<10	possible	0*	no	dead
8	60	M	no	N/A	N/A	13	no	dead
9	60	M	no	N/A	N/A	0*	carboplatin/paclitaxel	alive

AERP, acute, extensive radiation pneumonitis; pre-ILD, pre-existing interstitial lung disease; UIP, usual interstitial pneumonia; RT, radiotherapy; M, male; N/A, not applicable

*, AERP developed in the course of radiotherapy

The mean number of days to AERP development from the beginning of RT was 6.7 days. Five patients developed AERP in the middle of an RT, and they were forced to terminate RT, which resulted in a low RT intensity in the patients with AERP; on average, the patients with AERP received a total dose of 30 Gy, V20 and V30 were 7.9 and 4.7%, respectively, and the MLD was 4.3 Gy. These values were 39.7 Gy, 9.8%, 7.0%, and 5.7 Gy, respectively, in patients without AERP. For treating AERP, 7 patients required oral or intravenous administration of corticosteroids, and 3 patients received mechanical ventilation. Six patients with AERP died within 90 days of final irradiation, at least four of whom died of respiratory failure; however, the cause of death in the two remaining patients was unclear.

### Predictive factors of AERP

We analyzed predictive factors for AERP development using univariate and multivariate logistic regression models, and the results are shown in Table 3. The multivariate analysis revealed that the presence of ILD (odds ratio [OR] = 22.6; 95% confidence interval [CI] = 5.29–155; *p* < 0.001) and PS ≥ 2 (OR = 4.22; 95% CI = 1.06–20.8; *p* = 0.014) were significant predictive factors for the development of AERP. Further analysis was performed in patients with pre-ILD. Using univariate logistic regression, we found that ILD occupying more than 10% of the lung field (OR = 7.22; 95% CI = 1.43–40.8; *p* = 0.017) and CRP levels at the initiation of RT (OR = 35.3; 95% CI = 1.38–1241; *p* = 0.032) were significantly associated with the development of AERP (Table 4). Multivariate analysis of these 2 factors revealed that ILD involving more than 10% of the lung field in patients with pre-ILD was the only independent predictive factor (OR = 6.14; 95% CI = 1.07–37.4, *p* = 0.038) for the development of AERP.

**Table 3:** Univariate and Multivariate Logistic Analyses of the Risk of AERP in All Patients.

	Univariate Analysis		Multivariate Analysis	
	OR (95% CI)	*p-*value	OR (95% CI)	*p*-value
Age, years	0.58 (0.01–36.4)	0.782		
Pack-year smoking	12.5 (0.21–264)	0.153		
Concurrent ChT, yes	0.53 (0.07–2.19)	0.424		
PS, ≥2	5.37 (1.40–25.7)	0.014*	4.22 (1.06–20.8)	0.049*
Pre-ILD, yes	25.7 (6.08–174)	<0.001*	22.6 (5.29–155)	<0.001*
**Irradiated dose**				
Dose per fraction, Gy	0.01 (8.32–1.12)	0.208		
**Purpose of RT**				
Curative	0.36 (0.07–1.37)	0.150		
**Target organ**				
Lung/mediastinum	0.64 (0.16–3.07)	0.534		
**Pulmonary function**				
%FVC	0.42 (0.00–68.3)	0.730		
%FEV1.0	10.9 (0.19–685)	0.247		
**Laboratory findings**				
LDH, IU/l	13.7 (2.46–1006)	0.338		
CRP, mg/dl	29.2 (0.07–1501)	0.111		

*, *p* < 0.05

OR, odds ratio; CI, confidence interval; ChT, chemotherapy; PS, WHO performance status; pre-ILD, preexisting interstitial lung disease; RT, radiotherapy; FVC, forced vital capacity; FEV, forced expiratory volume; LDH, lactate dehydrogenase; CRP, C-reactive protein.

**Table 4:** Univariate and Multivariate Logistic Analyses of the Risk of AERP in Patients with pre-ILD.

	Univariate Analysis		Multivariate Analysis	
	OR (95%CI)	*p* Values	OR (95%CI)	*p* Values
Age, yr	0.22 (0.01–4.79)	0.330		
Pack-year smoking	2.11 (0.01–166)	0.756		
Concurrent ChT, yes	0.48 (0.02–3.02)	0.503		
PS, ≥2	4.90 (0.98–35.9)	0.068		
**Area occupied by pre-ILD**				
≥10%	7.22 (1.43–40.8)	0.017*	6.14 (1.0–37.4)	0.038*
**UIP compatibility of pre-ILD**				
Definite/possible UIP pattern	1.97 (0.10–14.6)	0.559		
**Irradiated dose**				
Dose per fraction, Gy	0.06 (0.00–1.84)	0.309		
**Purpose of RT**				
Curative	3.33 (0.67–24.3)	0.165		
**Target organ**				
Lung/mediastinum	2.40 (0.32–1.8)	0.355		
**Pulmonary function**				
%FVC	0.41 (0.01–21.6)	0.639		
%FEV1.0	3.30 (0.07–267)	0.562		
**Laboratory findings**				
LDH, IU/l	1.43 (0.00–152)	0.911		
CRP, mg/dl	35.2 (1.38–1,241)	0.032*	19.7 (0.59–1082)	0.108

*, *p* < 0.05

OR, odds ratio; CI, confidence interval; ChT, chemotherapy; PS, WHO performance status; pre-ILD, preexisting interstitial lung disease; UIP, usual interstitial pneumonia; RT, radiotherapy; FVC, forced vital capacity; FEV, forced expiratory volume; LDH, lactate dehydrogenase; CRP, C-reactive protein.

## Discussion

The current study illustrated that 1.4% of 651 RT targeting areas involving lung fields led to the development of AERP. The presence of pre-ILD and PS (≥2) at the beginning of RT were predictive of AERP, and furthermore, pre-ILD occupying more than 10% of the lung field increased the risk of AERP by 6-fold compared to pre-ILD occupying less than 10%. The UIP compatibility of pre-ILD was significantly associated with the risk of AERP.

Although several studies previously reported an association between pre-ILD and radiation pneumonitis [4,11,16,17,18,19,20,21,22], our current study is different regarding 2 points.

First, we analyzed patients with AERP. Previously, Morgan, et al. proposed 2 distinct forms of radiation pneumonitis; cRP and sporadic radiation pneumonitis [28]. cRP is caused by radiation-induced local cytokine production. It is confined to the irradiated area of the lung field, and it leads to fibrosis. Sporadic radiation pneumonitis is caused by an immunologically mediated process resulting in bilateral lymphocytic alveolitis that causes an “out-of-field” response to localized pulmonary irradiation, which resolves without sequelae. Sporadic radiation pneumonitis rarely causes radiological abnormality of the lungs; however, several cases of respiratory failure with bilateral “out-of-field” ground-glass opacity or infiltration have been reported, and the fatal ratio of reported cases was 7–25% despite aggressive treatments including corticosteroids [3,4,5,6,7,8,9,10,11]. According to these reports, AERP could be considered a severe case of sporadic radiation pneumonitis, and it is not surprising that AERP has its own predictive factors that differ from those of cRP.

Second, we explored the impact of CT findings of pre-ILD. Although the association between UIP compatibility and chemotherapy- or pulmonary resection-related lung injury was reported [25,29], little is known regarding RT. To our knowledge, only 1 study reported that the absence of the honeycombing was associated with an OR of 0.083 for symptomatic radiation pneumonitis, although the study only patients who received concurrent chemoradiotherapy [19]. In the current study, although patients with pre-ILD judged as having definite or possible UIP patterns exhibited a higher frequency of AERP (29%) than patients with ILD regarded as inconsistent with UIP (0%), UIP compatibility was not identified as a statistically significant predictive factor for AERP. On the contrary, the area occupied by pre-ILD, when it exceeded 10%, was revealed to be significantly associated with a risk of AERP (OR = 6.14; 95% CI = 1.07–37.4; *p* = 0.038). Kudoh et al. reported that a normal lung area on chest CT was associated with a risk of chemotherapy-related acute ILD [29]. However, to our knowledge, no previous studies investigated the association between the area occupied by pre-ILD and radiation-associated lung injury.

It was also noticeable that 4% of patients who received palliative RT developed AERP, including 2 patients who received RT targeting the thoracic spine, compared with only 1% of patients who underwent curative RT. It is unclear why patients who received palliative RT were more likely to develop AERP; however, our analysis illustrated that poor PS (≥2) was significantly associated with the risk of AERP, and patients who receive palliative RT are considered to have a poorer condition than those who receive curative RT. It has been reported that cancers associated with inflammatory cytokines including interleukin-6 are associated with deterioration of patient PS [30,31]. Together with the finding that CRP was significantly associated with AERP on univariate analysis in patients with pre-ILD, underlying inflammatory conditions may play a crucial role on the development of AERP.

Furthermore, although dosimetric parameters were not analyzed in the current study because of early termination of RT as a result of the development of AERP, the radiation dose was lower in patients who developed AERP than in those who did not develop AERP, which may indicate that the area or dose of irradiation might be less important for AERP development than for cRP [1,2].

Our study had some limitations. The presence of and area occupied by pre-ILD were determined semiquantitatively by the specialists, and the final decision was made depending on the discussion and majority. Recently, automated quantification of CT findings in pulmonary fibrosis was reported to be useful for survival prediction [32,33], and analysis with these more objective methods is expected in the future. The number of patients with AERP was small, and this could cause weak power for the detection of potential predictive factors. Possible differences related to racial or genetic background were not explored because most analyzed patients were of Japanese descent. Further studies with greater numbers of patients with AERP will be required to confirm the current results.

## Conclusion

A total of 1.4% of patients who underwent RT targeted to areas involving the lung field developed AERP. PS and pre-ILD, particularly when affecting more than 10% of the lung field, were associated with the risk of developing AERP. Pretreatment chest CT should be cautiously evaluated for the presence of and the area occupied by pre-ILD for safer irradiation.

## Supporting Information

S1 Table Individual data. (XLSX)

## References

[1] Fay M, Tan A, Fisher R, Mac Manus M, Wirth A, Ball D. Dose-volume histogram analysis as predictor of radiation pneumonitis in primary lung cancer patients treated with radiotherapy. Int J Radiat Oncol Biol Phys. 2005;61: 1355–1363. 15817337 10.1016/j.ijrobp.2004.08.025

[2] Rancati T, Ceresoli GL, Gagliardi G, Schipani S, Cattaneo GM. Factors predicting radiation pneumonitis in lung cancer patients: a retrospective study. Radiother Oncol. 2003;67: 275–283. 12865175 10.1016/s0167-8140(03)00119-1

[3] Smith JC. Radiation pneumonitis. Case report of bilateral reaction after unilaternal irradiation. Am Rev Respir Dis. 1964;89: 264–269. 14117688 10.1164/arrd.1964.89.2.264

[4] Makimoto T, Tsuchiya S, Hayakawa K, Saitoh R, Mori M. Risk factors for severe radiation pneumonitis in lung cancer. Jpn J Clin Oncol. 1999;29: 192–197. 10340042 10.1093/jjco/29.4.192

[5] Bennett DE, Million RR, Ackerman LV. Bilateral radiation penumonitis, a complication of the radiotherapy of bronchogenic carcinoma. (Report and analysis of seven cases with autopsy). Cancer. 1969;23: 1001–1018. 4976030 10.1002/1097-0142(196905)23:5<1001::aid-cncr2820230505>3.0.co;2-e

[6] Fulkerson WJ, McLendon RE, Prosnitz LR. Adult respiratory distress syndrome after limited thoracic radiotherapy. Cancer. 1986;57: 1941–1946. 3955500 10.1002/1097-0142(19860515)57:10<1941::aid-cncr2820571009>3.0.co;2-9

[7] Byhardt RW, Abrams R, Almagro U. The association of adult respiratory distress syndrome (ARDS) with thoracic irradiation (RT). Int J Radiat Oncol Biol Phys. 1988;15: 1441–1446. 3198441 10.1016/0360-3016(88)90241-6

[8] Pluzanska A, Chmielowska E, Stempczynska J, Alwasiak J, Wrezel B. [ARDS (adult respiratory distress syndrome) after chemotherapy and radiotherapy. Complications in two patients treated for non-Hodgkin's lymphoma]. Acta Haematol Pol. 1995;26: 219–225. 7653229

[9] Hwang JH, Lee KS, Song KS, Kim H, Kwon OJ, Lim TH, et al Extensive acute lung injury following limited thoracic irradiation: radiologic findings in three patients. J Korean Med Sci. 2000;15: 712–717. 11194201 10.3346/jkms.2000.15.6.712 PMC3054705

[10] Shi A, Zhu G, Wu H, Yu R, Li F, Xu B. Analysis of clinical and dosimetric factors associated with severe acute radiation pneumonitis in patients with locally advanced non-small cell lung cancer treated with concurrent chemotherapy and intensity-modulated radiotherapy. Radiat Oncol. 2010;5: 35 10.1186/1748-717X-5-35 20462424 PMC2883984

[11] Sanuki N, Ono A, Komatsu E, Kamei N, Akamine S, Yamazaki T, et al Association of computed tomography-detected pulmonary interstitial changes with severe radiation pneumonitis for patients treated with thoracic radiotherapy. J Radiat Res. 2012;53: 110–116. 22302051 10.1269/jrr.110142

[12] Hubbard R, Venn A, Lewis S, Britton J. Lung cancer and cryptogenic fibrosing alveolitis. A population-based cohort study. Am J Respir Crit Care Med. 2000;161: 5–8. 10619790 10.1164/ajrccm.161.1.9906062

[13] Park J, Kim DS, Shim TS, Lim CM, Koh Y, Lee SD, et al Lung cancer in patients with idiopathic pulmonary fibrosis. Eur Respir J. 2001;17: 1216–1219. 11491167 10.1183/09031936.01.99055301

[14] Turner-Warwick M, Lebowitz M, Burrows B, Johnson A. Cryptogenic fibrosing alveolitis and lung cancer. Thorax. 1980;35: 496–499. 7434310 10.1136/thx.35.7.496 PMC471320

[15] Ozawa Y, Suda T, Naito T, Enomoto N, Hashimoto D, Fujisawa T, et al Cumulative incidence of and predictive factors for lung cancer in IPF. Respirology. 2009;14: 723–728. 10.1111/j.1440-1843.2009.01547.x 19659650

[16] Robnett TJ, Machtay M, Vines EF, McKenna MG, Algazy KM, McKenna WG. Factors predicting severe radiation pneumonitis in patients receiving definitive chemoradiation for lung cancer. Int J Radiat Oncol Biol Phys. 2000;48: 89–94. 10924976 10.1016/s0360-3016(00)00648-9

[17] Leprieur E, Fernandez D, Chatellier G, Klotz S, Giraud P, Durdux C. Acute radiation pneumonitis after conformational radiotherapy for nonsmall cell lung cancer: Clinical, dosimetric, and associated-treatment risk factors. J. Cancer Res. Ther. 2013;9: 447 10.4103/0973-1482.119339 24125981

[18] Ueki N, Matsuo Y, Togashi Y, Kubo T, Shibuya K, Iizuka Y, et al Impact of pretreatment interstitial lung disease on radiation pneumonitis and survival after stereotactic body radiation therapy for lung cancer. J Thorac Oncol. 2015;10: 116–125. 10.1097/JTO.0000000000000359 25376512

[19] Tsujino K, Hashimoto T, Shimada T, Yoden E, Fujii O, Ota Y, et al Combined analysis of V20, VS5, pulmonary fibrosis score on baseline computed tomography, and patient age improves prediction of severe radiation pneumonitis after concurrent chemoradiotherapy for locally advanced non-small-cell lung cancer. J Thorac Oncol. 2014;9: 983–990. 10.1097/JTO.0000000000000187 24922010

[20] Takeda A, Ohashi T, Kunieda E, Sanuki N, Enomoto T, Takeda T, et al Comparison of clinical, tumour-related and dosimetric factors in grade 0–1, grade 2 and grade 3 radiation pneumonitis after stereotactic body radiotherapy for lung tumours. Br J Radiol. 2012;85: 636–642. 10.1259/bjr/71635286 22253343 PMC3479872

[21] Yamaguchi S, Ohguri T, Ide S, Aoki T, Imada H, Yahara K, et al Stereotactic body radiotherapy for lung tumors in patients with subclinical interstitial lung disease: the potential risk of extensive radiation pneumonitis. Lung Cancer. 2013;82: 260–265. 10.1016/j.lungcan.2013.08.024 24054547

[22] Liu H, Zhang X, Vinogradskiy YY, Swisher SG, Komaki R, Chang JY. Predicting radiation pneumonitis after stereotactic ablative radiation therapy in patients previously treated with conventional thoracic radiation therapy. Int J Radiat Oncol Biol Phys. 2012; 84: 1017–1023. 10.1016/j.ijrobp.2012.02.020 22543216 PMC3612879

[23] Kenmotsu H, Naito T, Kimura M, Ono A, Shukuya T, Nakamura Y, et al The risk of cytotoxic chemotherapy-related exacerbation of interstitial lung disease with lung cancer. J Thorac Oncol. 2011;6: 1242–1246. 10.1097/JTO.0b013e318216ee6b 21623239

[24] Travis WD, Costabel U, Hansell DM, King TE Jr., Lynch DA, Nicholson AG, et al An official American Thoracic Society/European Respiratory Society statement: Update of the international multidisciplinary classification of the idiopathic interstitial pneumonias. Am J Respir Crit Care Med. 2013;188: 733–748. 10.1164/rccm.201308-1483ST 24032382 PMC5803655

[25] Sugiura H, Takeda A, Hoshi T, Kawabata Y, Sayama K, Jinzaki M, et al Acute exacerbation of usual interstitial pneumonia after resection of lung cancer. Ann Thorac Surg. 2012;93: 937–943. 10.1016/j.athoracsur.2011.12.010 22305054

[26] Hansell DM, Bankier AA, MacMahon H, McLoud TC, Muller NL, Remy J. Fleischner Society: glossary of terms for thoracic imaging. Radiology. 2008;246: 697–722. 10.1148/radiol.2462070712 18195376

[27] Raghu G, Collard HR, Egan JJ, Martinez FJ, Behr J, Brown KK, et al An official ATS/ERS/JRS/ALAT statement: idiopathic pulmonary fibrosis: evidence-based guidelines for diagnosis and management. Am J Respir Crit Care Med. 2011;183: 788–824. 10.1164/rccm.2009-040GL 21471066 PMC5450933

[28] Morgan GW, Breit SN. Radiation and the lung: a reevaluation of the mechanisms mediating pulmonary injury. Int J Radiat Oncol Biol Phys. 1995;31: 361–369. 7836090 10.1016/0360-3016(94)00477-3

[29] Kudoh S, Kato H, Nishiwaki Y, Fukuoka M, Nakata K, Ichinose Y, et al Interstitial lung disease in Japanese patients with lung cancer: a cohort and nested case-control study. Am J Respir Crit Care Med. 2008;177: 1348–1357. 10.1164/rccm.200710-1501OC 18337594

[30] Scott HR, McMillan DC, Forrest LM, Brown DJ, McArdle CS, Milroy R. The systemic inflammatory response, weight loss, performance status and survival in patients with inoperable non-small cell lung cancer. Br J Cancer. 2002;87: 264–267. 12177792 10.1038/sj.bjc.6600466 PMC2364225

[31] Martin F, Santolaria F, Batista N, Milena A, Gonzalez-Reimers E, Brito MJ, et al Cytokine levels (IL-6 and IFN-gamma), acute phase response and nutritional status as prognostic factors in lung cancer. Cytokine. 1999;11: 80–86. 10080883 10.1006/cyto.1998.0398

[32] Maldonado F, Moua T, Rajagopalan S, Karwoski RA, Raghunath S, Decker PA, et al Automated quantification of radiological patterns predicts survival in idiopathic pulmonary fibrosis. Eur Respir J. 2014;43: 204–212. 10.1183/09031936.00071812 23563264

[33] Rosas IO, Yao J, Avila NA, Chow CK, Gahl WA, Gochuico BR. Automated quantification of high-resolution CT scan findings in individuals at risk for pulmonary fibrosis. Chest. 2011;140: 1590–1597. 10.1378/chest.10-2545 21622544 PMC3231958

